# Personalized prediction of esophageal cancer risk based on virtually generated alcohol data

**DOI:** 10.1186/s12967-025-06383-9

**Published:** 2025-03-28

**Authors:** Oswald Ndi Nfor, Pei-Ming Huang, Ming-Fang Wu, Ke-Cheng Chen, Ying-Hsiang Chou, Mong-Wei Lin, Ji-Han Zhong, Shuenn-Wen Kuo, Yu-Kwang Lee, Chih-Hung Hsu, Jang-Ming Lee, Yung-Po Liaw

**Affiliations:** 1 Department of Public Health, Institute of Public Health, Chung Shan Medical University, No.110, Sec.1, Jianguo North Road, Taichung, 40201 Taiwan; 2 Department of Medicine, National Taiwan University College of Medicine, No.1, Sec.1, Jen-Ai Road, Taipei, 100233 Taiwan; 3 Division of Thoracic Surgery, Department of Surgery, National Taiwan University Hospital, No.7, Chung-Shan South Road, Taipei, 100225 Taiwan; 4 School of Medicine, Chung Shan Medical University, No. 110, Sec. 1, Jianguo North Road, 40201 Taichung, Taiwan; 5 Divisions of Medical Oncology and Chest Medicine, Chung Shan Medical University Hospital, No. 110, Sec. 1, Jianguo Nord Road, 40201 Taichung, Taiwan; 6 Department of Radiation Oncology, Chung Shan Medical University Hospital, No. 110, Sec. 1, Jianguo Nord Road, 40201 Taichung, Taiwan; 7 School of Medical Imaging and Radiological Sciences, Chung Shan Medical University, No. 110, Sec. 1, Jianguo Nord Road, 40201 Taichung, Taiwan; 8 Division of General Surgery, Department of Surgery, National Taiwan University Hospital, No.7, Chung-Shan South Road, Taipei, 100225 Taiwan; 9 Department of Medical Oncology, National Taiwan University Cancer Center, No. 57, Lane 155, Section 3, Keelung Road, Taipei, 106, Taiwan; 10 Department of Oncology, National Taiwan University Hospital, No.7, Chung Shan South Road, Taipei, 100225 Taiwan; 11 Graduate Institute of Oncology, National Taiwan University College of Medicine, No.1, Sec.1, Jen-Ai Road, Taipei, 100233 Taiwan; 12 Department of Medical Imaging, Chung Shan Medical University Hospital, No.110, Sec.1, Jianguo North Road, Taichung, 402306 Taiwan; 13 Institute of Medicine, Chung Shan Medical University, No.110, Sec.1, Jianguo North Road, Taichung, 402306 Taiwan

**Keywords:** Predictive medicine, Personalized medicine, Cancers, Esophagus, Risk assessment

## Abstract

**Background:**

Esophageal cancer (EC) presents a significant public health challenge globally, particularly in regions with high alcohol consumption. Its etiology is multifactorial, involving both genetic predispositions and lifestyle factors.

**Methods:**

This study aimed to develop a personalized risk prediction model for EC by integrating genetic polymorphisms (rs671 and rs1229984) with virtually generated alcohol consumption data, utilizing advanced artificial intelligence and machine learning techniques. We analyzed data from 86,845 individuals, including 763 diagnosed EC patients, sourced from the Taiwan Biobank. Eight machine learning models were employed: Bayesian Network, Decision Tree, Ensemble, Gradient Boosting, Logistic Regression, LASSO, Random Forest, and Support Vector Machines (SVM). A unique aspect of our approach was the virtual generation of alcohol consumption data, allowing us to evaluate risk profiles under both consuming and non-consuming scenarios.

**Results:**

Our analysis revealed that individuals with the genotypes rs671 = AG and rs1229984 = CC exhibited the highest probabilities of developing EC, with values ranging from 0.2041 to 0.9181. Notably, abstaining from alcohol could decrease their risk by approximately 16.29–49.58%. The Ensemble model demonstrated exceptional performance, achieving an area under the curve (AUC) of 0.9577 and a sensitivity of 0.9211. This transition from consumption to abstinence indicated a potential risk reduction of nearly 50% for individuals with high-risk genotypes.

**Conclusion:**

Overall, our findings highlight the importance of integrating virtually generated alcohol data for more precise personalized risk assessments for EC.

## Introduction

Esophageal cancer (EC) poses a significant public health concern globally [1], particularly in regions with high alcohol consumption [2]. The disease’s etiology is multifaceted, involving genetic predispositions and lifestyle factors. Among the genetic factors, single nucleotide polymorphisms (SNPs) in the alcohol dehydrogenase (*ADH*) and aldehyde dehydrogenase (*ALDH*) genes, specifically rs671 and rs1229984, have been shown to significantly influence disease risk, particularly esophageal squamous cell carcinoma (ESCC) [3–5]. These genetic variants affect the metabolism of ethanol and its toxic byproduct, acetaldehyde, a known carcinogen linked to various cancers, including EC [6, 7].

The *ALDH2* rs671 and *ADH1B* rs1229984 polymorphisms are recognized for their significant associations with alcohol metabolism and related health outcomes. When comparing the prevalence and impacts of these genetic variants across global populations, notable differences arise, particularly between Eastern and Western ancestry groups. The rs671 polymorphism is especially prevalent in East Asian populations. A meta-analysis indicated that approximately 50% of East Asians carry at least one copy of the inactive A allele, leading to severely reduced enzyme function [8]. In contrast, this polymorphism is virtually absent in European populations, where the G allele is predominant, leading to a stark contrast in alcohol metabolism and potential alcohol-related health risks across these groups [9].

Conversely, the rs1229984 polymorphism in the *ADH1B* gene exhibits a different prevalence pattern. This variant is more commonly found in populations of European descent, with a frequency of about 26% among East Asians compared to about 40% in certain European populations [10]. While rs1229984 has a weaker impact on alcohol consumption compared to rs671, it still plays a significant role in modulating alcohol-related health risks, particularly in conjunction with other risk factors [11]. Population studies illustrate that the combined effects of *ALDH2* rs671 and *ADH1B* rs1229984 vary significantly; for instance, individuals carrying the dysfunctional rs671 variant alongside the *ADH1B* rs1229984 variant experience heightened risks of developing alcohol-related diseases, including various cancers in East Asian populations [10, 12].

Recent studies have emphasized the importance of understanding gene-environment interactions contributing to EC risk. Specifically, the presence of the *ALDH2* rs671 variant has been associated with increased susceptibility to EC in East Asians, particularly when combined with high alcohol consumption [13, 14]. A genotype-stratified genome-wide association study (GWAS) identified several variants, including rs671 and rs1229984, that significantly impact the risk of EC in Japanese populations, underscoring the relevance of personalized genetic assessments in predicting cancer risk [15]. This approach aligns with the growing emphasis on precision public health, which seeks to tailor prevention strategies based on individual genetic profiles, lifestyle factors, and clinical characteristics [16, 17].

The rationale for focusing on personalized prediction of EC is underscored by the high prevalence of the ALDH2*2 allele among East Asians, which can lead to a dramatic increase in cancer risk among heavy drinkers [18, 19]. For instance, it has been estimated that if moderate-to-heavy alcohol consumers with the ALDH2*1/*2 genotype reduced their consumption to lower levels, it could potentially reduce EC cases by 53% in Japanese men [20]. This highlights the potential for targeted public health interventions that incorporate genetic screening to identify individuals at high risk and promote lifestyle modifications to mitigate their risk [11, 21].

Further, the integration of insights from recent research emphasizes the dynamic interplay between cellular immunity and tumor cells in cancer development, suggesting that a comprehensive understanding of these interactions can inform prevention strategies. For example, Aghapour et al. (2024) highlight the critical role of the immune response in modulating tumor behavior, suggesting that personalized interventions could leverage this relationship effectively [22].

So far, most of the studies assessing disease risk have relied on traditional research methods. The utilization of virtually generated data in assessing disease risk, particularly in the context of EC and its association with genetic variants such as rs671 and rs1229984, presents several advantages over traditional epidemiological research that often relies on control data from disparate sources. One of the primary benefits of virtually generated data is the ability to create a controlled environment where variables can be systematically manipulated and analyzed, leading to more precise and reliable conclusions regarding the interactions between genetic predispositions and lifestyle factors, such as alcohol consumption.

Many previous studies have often used conventional methods like logistic regression. However, with advancements in technology, AI and machine learning now offer powerful tools to analyze large datasets and uncover patterns, helping to improve our understanding of EC screening, monitoring, and treatment [23]. Their application in the assessment and diagnosis of EC represents a significant advancement in the field of oncology [24]. Machine learning models have also been developed to predict the five-year survival status of EC patients based on clinical data [25]. These models leverage several features, including demographic and clinical variables, to provide personalized prognostic information that can guide treatment decisions. In light of this, we incorporated genetic, lifestyle, and virtual alcohol data to assess EC cancer risk among biobank participants with rs671 and rs1229984 polymorphisms using machine learning models.

## Materials and methods

### Study population/disease information

**Fig. 1:**
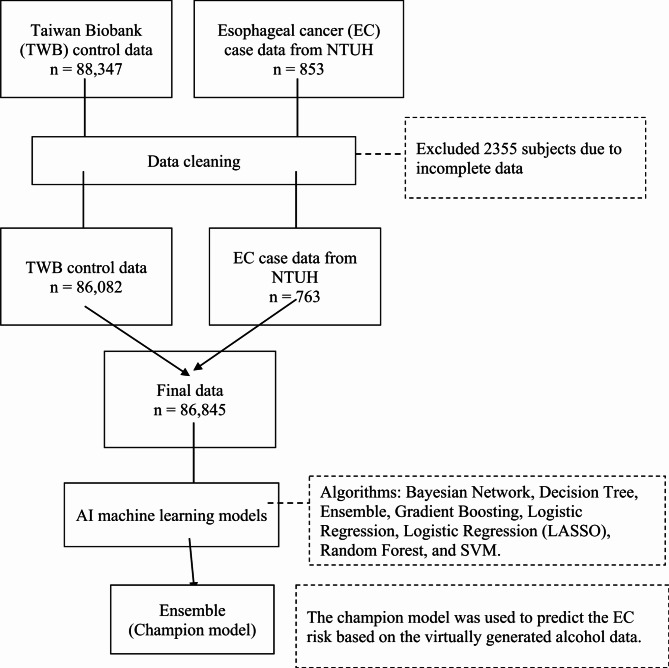
Data processing pipeline

This study utilized data from two primary sources: the TWB (control data) and the National Taiwan University Hospital (case data). TWB participants provided informed consent before their data were collected. Overall, data were available for 89,200 individuals from both data sources. Exclusion criteria included 2355 individuals with incomplete data (Fig. 1). Consequently, the total sample size encompassed 86,845 participants, comprising 86,082 controls and 763 EC patients. Ethics approval was obtained from the Institutional Review Board of Chung Shan Medical University Hospital (No. CS2-21160). All procedures adhered to the ethical standards established by the responsible committee on human experimentation, as well as the Helsinki Declaration of 1964 and its later versions. Individuals from the Taiwan Biobank provided written informed consent during enrollment.

Patients diagnosed with primary EC were included as cases. Demographic and lifestyle factors, including sex, age, cigarette smoking, alcohol drinking, and betel nut chewing, were defined using self-reported responses to TWB questionnaires. Cigarette smokers were defined as those who had smoked or regularly smoked for at least six months. Alcohol consumers were defined as those who had consumed or regularly consumed at least 150 cc of alcohol per week for at least six months.

### SNP genotyping and imputation

Whole-genome genotyping was performed using the Axiom Genome-Wide Array Plate chip system (Affymetrix Inc., Santa Clara, CA, USA) TWB (V2.0) chip. Candidate SNPs, including *ALDH2* rs671, *HECTD4* rs2074356, *ADH1B* rs1042026 and rs1229984, *GSTP1* rs1695, *ERCC5* rs17655, *PTGS2* rs20417, *XRCC1* rs25487, *MTHFR* rs1801133, *ADH4* rs3805322, *PLCE1* rs2274223 and rs3765524, rs11066015, and rs11066280, *PDE4D* rs10052657, *RUNX1* rs2014300, and *SLC39A6* rs1050631, were genotyped. Some SNPs, including *CYP1A1* rs1048943, *CISH* rs2239751, *SOCS1* rs243324, *ERCC2* rs238406, and *PLCE1* rs7922612, were imputed using TWB’s genotype imputation process [11, 26–36].

### Statistical analyses and machine learning models

Demographic data distributions were assessed using chi-square tests for categorical variables, with results presented as counts and percentages. Data management and analysis were conducted using SAS 9.4 (SAS Institute, Cary, NC, USA) and PLINK 1.90 beta (Shaun Purcell & Christopher Chang). A significance threshold was set at *P* < 0.05.

To develop the machine learning models, we utilized SAS^®^ Viya^®^ (version 3.5, SAS Institute Inc., Cary, NC, USA). Our approach incorporated various supervised learning models, including Bayesian Networks, Decision Trees, Ensemble methods, Gradient Boosting, Logistic Regression (including LASSO), Random Forest, and Support Vector Machines (SVM). The dataset of 86,845 participants was randomly divided into training (60%), validation (30%), and testing (10%) subsets, with the target variable being EC. Input features comprised the 22 SNPs along with demographic covariates. Model performance was evaluated using the area under the curve of the receiver operating characteristic (AUC-ROC), sensitivity, and specificity. The KS (Youden) index, generated by SAS Viya, was employed to select the best predictive model among the candidates.

In the subsequent phase of this research, the methodology was adapted to incorporate virtually generated data, while maintaining the same sample size from both data sources. We created a virtual dataset that mirrored the original sample size while altering the drinking status of participants. The process involved the following steps:

#### Identification of drinking status

We categorized participants into two groups based on their reported alcohol consumption: consumers and non-consumers.

#### Modification of drinking status

To create the virtual dataset, we systematically altered the consumption status of each participant: For instance, participants classified as consumers were reclassified as abstainers (non-consumers). Conversely, those classified as abstainers were reclassified as consumers.

Importantly, all other demographic and health-related variables in the dataset remained unchanged. This allowed us to isolate the effect of altered alcohol consumption patterns while controlling for confounding factors that could influence EC risk. Based on the 10% test data performance, and using the champion model, we conducted personalized predictions on both the original and virtually generated data, enabling the assessment of individual probabilities for developing EC under varying conditions. This approach facilitated the calculation of increased risk associated with alcohol consumption for each participant.

## Results

The distribution of genotypes and alcohol consumption patterns among EC cases and controls is detailed in Table 1. Significant differences were observed between the two groups (*p* < 0.001). Notably, the highest percentage of EC patients (35.87%) was found in the subgroup carrying the rs671 = AG and rs1229984 = CC genotypes, along with a history of alcohol consumption. This was followed by the subgroup with the rs671 = AG and rs1229984 = CT genotypes, which accounted for 18.88% of EC patients. Another subgroup with a relatively high percentage of EC patients (6.84%) was identified as having the rs671 = GG and rs1229984 = CC genotypes, along with a consumption history.

**Table 1:** Descriptive data of the study participants based on the original dataset

Variables	Controls (a)	EC patients (b)	[b / (a + b)] *100
rs671 = GG, rs1229984 = TT, abstinence	21,367	25	0.12
rs671 = GG, rs1229984 = TT, consumption	2682	80	2.90
rs671 = GG, rs1229984 = CT, abstinence	15,294	18	0.12
rs671 = GG, rs1229984 = CT, consumption	2017	61	2.94
rs671 = GG, rs1229984 = CC, abstinence	2803	8	0.28
rs671 = GG, rs1229984 = CC, consumption	422	31	6.84
rs671 = AG, rs1229984 = TT, abstinence	18,069	55	0.30
rs671 = AG, rs1229984 = TT, consumption	872	138	13.66
rs671 = AG, rs1229984 = CT, abstinence	12,649	32	0.25
rs671 = AG, rs1229984 = CT, consumption	666	155	18.88
rs671 = AG, rs1229984 = CC, abstinence	2252	27	1.18
rs671 = AG, rs1229984 = CC, consumption	211	118	35.87
rs671 = AA, rs1229984 = TT, abstinence	3549	4	0.11
rs671 = AA, rs1229984 = TT, consumption	22	3	12.00
rs671 = AA, rs1229984 = CT, abstinence	2689	4	0.15
rs671 = AA, rs1229984 = CT, consumption	12	3	20.00
rs671 = AA, rs1229984 = CC, abstinence	504	1	0.20
rs671 = AA, rs1229984 = CC, drinking	2	0	0
**p-value**	< 0.001		

EC = esophageal cancer

Note: Abstinence refers to individuals who refrained from consuming alcohol, whereas consumption pertains to those who consumed alcohol

Across various genotype combinations, subgroups with a history of alcohol consumption generally demonstrated higher percentages of EC patients compared to those who abstained. Conversely, groups with the rs671 = AA genotype had fewer alcohol consumers in both EC cases and controls, regardless of the rs1229984 genotype, suggesting a potential self-protective mechanism in the body.

Table 2 presents a comparison of the performance of various machine learning algorithms evaluated on the testing dataset, which comprised 10% of the original data. The Ensemble model achieved the highest Youden’s J statistic (KS) of 0.8560, indicating excellent discrimination ability between positive and negative cases. It also attained the highest AUC of 0.9577, suggesting outstanding classification performance. Furthermore, the Ensemble classifier demonstrated high sensitivity (0.9211) and specificity (0.9349), reflecting its excellent ability to correctly identify positive and negative cases, respectively. The overall accuracy of the Ensemble model was 0.9348, indicating a high percentage of correct predictions.

**Table 2:** A comparison of the models evaluated on the testing dataset, which constituted 10% of the original data

Algorithm	KS (Youden)	AUC	Sen	Spe	Accuracy
Ensemble	0.8560	0.9577	0.9211	0.9349	0.9348
Bayesian Network	0.8014	0.9119	0.8421	0.9593	0.9583
SVM	0.7793	0.9374	0.8421	0.9372	0.9363
Random Forest	0.7345	0.8729	0.7632	0.9713	0.9695
Decision Tree	0.7247	0.8664	0.8026	0.9220	0.9210
Logistic Regression	0.7246	0.8623	0.8026	0.9219	0.9209
Logistic Regression (LASSO)	0.7035	0.8550	0.7368	0.9667	0.9646
Gradient Boosting	0.5700	0.7850	0.5789	0.9911	0.9874

Abbreviation: Sen = sensitivity, Spe = specificity, AUC = area under the curve, SVM = support vector machine, LASSO = Least Absolute Shrinkage and Selection Operator

The Bayesian Network algorithm also exhibited strong performance, with a KS of 0.8014 and an AUC of 0.9119. It achieved a high specificity of 0.9593, indicating a low false-positive rate, and an overall accuracy of 0.9583. The Support Vector Machine (SVM) algorithm attained a KS of 0.7793 and an AUC of 0.9374, demonstrating equal sensitivity and specificity of 0.8421, with an overall accuracy of 0.9363. Other algorithms, such as Random Forest, Decision Tree, and Logistic Regression, exhibited decent performance, with varying trade-offs between sensitivity, specificity, and overall accuracy. The Gradient Boosting algorithm had the lowest performance among the evaluated models, with a KS of 0.5700, an AUC of 0.7850, and a sensitivity of 0.5789; however, it achieved the highest specificity of 0.9911.

Table 3 displays the predictive probabilities for various genotype combinations and alcohol consumption statuses. As the champion model in this study, the Ensemble model’s predictive probabilities were utilized for the analysis of the original data. We categorized all participants into 18 groups based on the ADH1B rs671 and ALDH2 rs1229984 SNPs and alcohol consumption statuses. The predictive probabilities were presented as minimum (Min), lower quartile (Q1), median, mean, upper quartile (Q3), and maximum (Max) values. Among these groups, alcohol consumers with the rs671 = AG and rs1229984 = CC genotypes exhibited the highest predictive probability of EC. This group comprised 329 individuals, including 211 controls and 118 EC patients. In this group, the predictive probabilities of EC ranged from 0.2041 to 0.9181, with a median of 0.4751.

**Table 3:** The AI/ML predictive probabilities of EC in the ensemble model based on rs671 and rs1229984 genotypes and alcohol consumption data derived from the original dataset

Variables	Controls (*n*)	EC patients (*n*)	Predictive probability					
			Min	Q1	Median	Mean	Q3	Max
**Combination of SNPs and consumption pattern**								
rs671 = GG, rs1229984 = TT, abstinence	21,367	25	0.0324	0.1026	0.1252	0.1676	0.2720	0.4239
rs671 = GG, rs1229984 = TT, consumption	2682	80	0.1356	0.3497	0.4128	0.3704	0.4401	0.5438
rs671 = GG, rs1229984 = CT, abstinence	15,294	18	0.0417	0.1058	0.1282	0.1706	0.2740	0.4109
rs671 = GG, rs1229984 = CT, consumption	2017	61	0.1310	0.3535	0.4149	0.3727	0.4416	0.5328
rs671 = GG, rs1229984 = CC, abstinence	2803	8	0.0526	0.1221	0.1445	0.1838	0.1919	0.4239
rs671 = GG, rs1229984 = CC, consumption	422	31	0.1500	0.2545	0.4319	0.3811	0.4595	0.6339
rs671 = AG, rs1229984 = TT, abstinence	18,069	55	0.0623	0.1334	0.1569	0.2064	0.3187	0.4502
rs671 = AG, rs1229984 = TT, consumption	872	138	0.1753	0.4180	0.4525	0.4213	0.4728	0.7589
rs671 = AG, rs1229984 = CT, abstinence	12,649	32	0.0701	0.1367	0.1594	0.2071	0.3174	0.4389
rs671 = AG, rs1229984 = CT, consumption	666	155	0.1842	0.4209	0.4560	0.4268	0.4771	1.0000
rs671 = AG, rs1229984 = CC, abstinence	2252	27	0.1003	0.1525	0.1748	0.2172	0.3176	0.4494
rs671 = AG, rs1229984 = CC, consumption	211	118	0.2041	0.4482	0.4751	0.4738	0.4971	0.9181
rs671 = AA, rs1229984 = TT, abstinence	3549	4	0.0000	0.0661	0.0905	0.1414	0.2552	0.3661
rs671 = AA, rs1229984 = TT, consumption	22	3	0.1482	0.3214	0.3813	0.3476	0.4091	0.4515
rs671 = AA, rs1229984 = CT, abstinence	2689	4	0.0048	0.0702	0.0942	0.1463	0.2621	0.3845
rs671 = AA, rs1229984 = CT, consumption	12	3	0.1667	0.2173	0.3841	0.3305	0.4166	0.4477
rs671 = AA, rs1229984 = CC, abstinence	504	1	0.0352	0.0893	0.1155	0.1705	0.2882	0.4142
rs671 = AA, rs1229984 = CC, consumption	2	0	0.1483	0.1483	0.2860	0.2860	0.4237	0.4237

Adjusted for sex, age, cigarette smoking, betel nut chewing, and additive model of 20 SNPs, including rs1042026, rs1695, rs17655, rs20417, rs25487, rs1801133, rs3805322, rs2274223, rs3765524, rs2074356, rs11066015, rs11066280, rs10052657, rs2014300, rs1050631, rs1048943, rs2239751, rs243324, rs238406, rs7922612

Note: Abstinence refers to individuals who refrained from consuming alcohol, whereas consumption pertains to those who consumed alcohol

Table 4 (models comprising the original and virtual alcohol data) illustrates that if the 329 participants with rs671 = AG, rs1229984 = CC, and who currently consume alcohol were to abstain, their highest risk for EC could be reduced from 0.9181 to 0.4629, and their lowest risk could decrease from 0.2041 to 0.1227. If these individuals had never consumed alcohol, the probability of developing EC could decrease by as much as 0.4552 and as little as 0.0814. This indicates that if these individuals had never consumed alcohol, the percentage reduction in EC risk would range from 16.29 to 49.58%.

**Table 4:** Personalized predictions of EC risk in the ensemble model based on the original and virtual alcohol data, in conjunction with the genotypes rs671 = AG and rs1229984 = CC

	Predictive probability					
	Min	Q1	Median	Mean	Q3	Max
**rs671 = AG**,** rs1229984 = CC**,** consumption (original data**, *n* **= 329)**	0.2041	0.4482	0.4751	0.4738	0.4971	0.9181
**rs671 = AG**,** rs1229984 = CC**,** abstinence (virtual data**, *n* **= 329)**	0.1227	0.3668	0.3937	0.3654	0.4157	0.4629
**P (consumption– abstinence) (***n* **= 329)**	0.0814	0.0814	0.0814	0.1084	0.0814	0.4552
[P (consumption– abstinence)/ P consumption] *100 (*n* **= 329)**	16.2870	17.0006	17.8696	22.5879	28.8007	49.5790

Adjusted for sex, age, cigarette smoking, betel nut chewing, and additive model of 20 SNPs, including rs1042026, rs1695, rs17655, rs20417, rs25487, rs1801133, rs3805322, rs2274223, rs3765524, rs2074356, rs11066015, rs11066280, rs10052657, rs2014300, rs1050631, rs1048943, rs2239751, rs243324, rs238406, rs7922612

The analysis included data from a control group of 211 individuals and 118 patients diagnosed with EC, utilizing both original data and virtually generated alcohol data

## Discussion

This study is pioneering in its application of artificial intelligence (AI) and machine learning tools for the personalized prediction of EC risk. It integrates genetic factors related to alcohol metabolism with virtually generated alcohol consumption data. The results indicate significant predictive probabilities across various genotype and alcohol consumption status combinations. Notably, participants with the genotype combination of rs671 = AG, rs1229984 = CC, and alcohol consumption exhibited the highest predicted risk of developing EC.

The incorporation of virtually generated alcohol data is particularly crucial in this research. By simulating changes in consumption status, we assessed the potential impact of alcohol abstinence on EC risk, enhancing our understanding of how lifestyle modifications can influence health outcomes. This approach aligns with the growing recognition of the importance of virtual data in precision public health, which emphasizes personalized interventions based on individual risk factors [37, 38]. The ability to manipulate consumption status in our model allowed us to demonstrate that changing from “consumption” to “abstinence” could lead to a substantial reduction in predictive probabilities for individuals with high-risk genotypes. This highlights the significant role of alcohol consumption in modulating EC risk.

Considering the hypothesis proposed by previous research [3], which suggests that the A allele of rs671 and the T allele of rs1229984 are associated with reduced alcohol consumption, participants with rs671 = GG, rs1229984 = CC, and alcohol consumption were expected to have a higher predicted risk. However, our study’s predictive results differed somewhat from this hypothesis. Our findings revealed that participants with rs671 = AG, rs1229984 = CC, with a history of alcohol consumption, had the highest predicted risk, ranging from 0.2041 to 0.9181, with a median of 0.4751. In contrast, participants with rs671 = GG, rs1229984 = CC, and a history of alcohol consumption had a risk ranging from 0.1500 to 0.6339, with a median of 0.4319. Although this group did not have the highest risk of EC, they still represented a cohort with elevated risk. Among alcohol consumers, those with rs671 = AG exhibited higher counts and proportions of EC cases compared to the other six groups, regardless of their rs1229984 genotypes.

Conversely, case and control groups with rs671 = AA had fewer alcohol consumers, irrespective of the rs1229984 genotype. This is likely due to flushing reactions experienced by individuals with the rs671-A allele, caused by the accumulation of acetaldehyde, which influences drinking behaviors and decreases the likelihood of alcohol dependence [39]. Therefore, this genotype’s avoidance of the carcinogen acetaldehyde can be considered a self-protective mechanism.

Furthermore, we predicted the status of the 329 individuals with the highest predictive probabilities by changing their original “consumption” status to “abstinence”. Results showed that, while holding genotype and other factors constant, this change caused a decrease in the predictive probabilities for individuals with the genotype rs671 = GA and rs1229984 = CC from 0.1127 to 0.4629, with a median of 0.3937. This represents a substantial decrease, with the highest reduction of nearly 50%. This underscores the significant impact of alcohol consumption on EC risk, particularly for those with the genotype combination rs671 = GA and rs1229984 = CC.

Considering the median values, alcohol drinkers with the rs671-GG or AG genotype exhibited higher predicted risks of EC, regardless of their rs1229984 genotype, with all medians exceeding 0.4. In contrast, alcohol drinkers with rs671-AA, had lower risks of EC, with all medians below 0.4. This suggests that the A allele of rs671 has a greater impact on disease risk than the T allele of rs1229984. Individuals with the rs671-AA genotype showed a significant reduction in EC risk, aligning with a previous meta-analysis [40].

In our study, we analyzed the impact of alcohol consumption on EC risk by creating a virtual dataset that mirrored the original sample size while altering participants’ alcohol consumption statuses. This approach maintains the same sample size and demographic characteristics, ensuring robust comparisons between consumers and abstainers. It also allows for simulating a range of scenarios regarding alcohol consumption, providing insights into how variations in drinking behavior could influence cancer risk.

However, while the utilization of virtual data presents unique advantages, it also introduces potential biases due to design assumptions. For instance, reclassifying consumers to non-consumers and vice versa is based on the assumption that these classifications accurately capture diverse patterns of alcohol consumption. This simplification may overlook important nuances, such as variations in drinking frequency, quantity, and context, which can significantly influence health outcomes. Moreover, the assumptions underlying our virtual data generation raise critical considerations for real-world applications. One key assumption is that the effects of alcohol on EC risk are consistent across different populations and contexts, which may not hold true due to cultural, genetic, and environmental factors.

The implications of these assumptions for real-world applications are substantial. While our approach provides a valuable framework for exploring hypothetical scenarios of alcohol consumption, it is essential to interpret the results with caution. The findings derived from virtually generated data should be validated against empirical data and considered as part of a broader context that includes diverse population characteristics and behaviors. A concerted effort towards inclusive and representative research practices is essential to leverage the true potential of virtual data in improving public health and scientific understanding.

## Conclusions

In conclusion, our study establishes that individuals with the combination of rs671 = AG, rs1229984 = CC, and alcohol consumption face a heightened probability of developing EC. If these individuals were to abstain from drinking, their risk could be reduced by nearly 50%. The integration of virtually generated alcohol data into our predictive model significantly enhances personalized risk assessments for EC, demonstrating the transformative potential of AI and machine learning in advancing precision public health initiatives. While the virtual data generation method may appear unconventional, it serves as a valuable tool for understanding the complex relationship between alcohol consumption and EC risk. Our model is designed to be adaptable for use in various populations and health systems through effective strategies such as data integration, systematic calibration and validation, and cultural sensitivity. Collaborating with local health authorities and incorporating relevant demographic and consumption data will ensure the model’s applicability and predictive accuracy. These efforts will enhance the model’s scalability, making it a valuable tool for addressing the health implications of alcohol consumption across diverse contexts. We believe this approach enriches our study’s contributions to the field and provides a foundation for future research.

## Data Availability

The data supporting the findings of this study are protected and cannot be made publicly available. They are however available from the corresponding author (Yung-Po Liaw) upon reasonable request and with permission of Taiwan Biobank.

## Abbreviations

EC: Esophageal cancer
AUC: Area under the curve
BMI: Body mass index
OR: Odds ratio
CI: 95% confidence interval
ALDH: Aldehyde dehydrogenase
TWB: Taiwan biobank
ADH: Alcohol dehydrogenase
ESCC: Squamous cell carcinoma
SVM: Support vector machines
